# Solid-Phase Extraction and Reverse-Phase HPLC: Application to Study the Urinary Excretion Pattern of Benzophenone-3 and its Metabolite 2,4-Dihydroxybenzophenone in Human Urine

**DOI:** 10.4137/aci.s396

**Published:** 2008-01-31

**Authors:** Helena Gonzalez, Carl-Eric Jacobson, Ann-Marie Wennberg, Olle Larkö, Anne Farbrot

**Affiliations:** 1 Department of Dermatology and Venereology, Sahlgrenska University Hospital, S-413 45 Göteborg, Sweden; 2 Department of Clinical Chemistry/Transfusion Medicine, Sahlgrenska University Hospital, S-413 45 Göteborg, Sweden; 3 Current address SCA

**Keywords:** benzophenone-3, 2,4-dihydroxybenzophenone, HPLC, reverse-phase HPLC, oxybenzone, sunscreen

## Abstract

**Background:**

Benzophenone-3 (BZ-3) is a common ultraviolet (UV) absorbing compound in sunscreens. It is the most bioavailable species of all UV-absorbing compounds after topical application and can be found in plasma and urine.

**Objectives:**

The aim of this study was to develop a reverse-phase high performance liquid chromatography (HPLC) method for determining the amounts BZ-3 and its metabolite 2,4-dihydroxybenzophenone (DHB) in human urine. The method had to be suitable for handling a large number of samples. It also had to be rapid and simple, but still sensitive, accurate and reproducible. The assay was applied to study the urinary excretion pattern after repeated whole-body applications of a commercial sunscreen, containing 4% BZ-3, to 25 healthy volunteers.

**Methods:**

Each sample was analyzed with regard to both conjugated/non-conjugated BZ-3 and conjugated/non-conjugated DHB, since both BZ-3 and DHB are extensively conjugated in the body. Solid-phase extraction (SPE) with C8 columns was followed by reverse-phase HPLC. For separation a Genesis C18 column was used with an acethonitrile-water mobile phase and the UV-detector was set at 287 nm.

**Results:**

The assay was linear *r*^2^ > 0.99, with detection limits for BZ-3 and DHB of 0.01 μmol L^−1^ and 0.16 μmol L^−1^ respectively. Relative standard deviation (RSD) was less than 10% for BZ-3 and less than 13% for DHB. The excretion pattern varied among the human volunteers; we discerned different patterns among the individuals.

**Conclusions:**

The reverse-phase HPLC assay and extraction procedures developed are suitable for use when a large number of samples need to be analyzed and the method fulfilled our objectives. The differences in excretion pattern may be due to differences in enzyme activity but further studies, especially about genetic polymorphism, need to be performed to verify this finding.

## Introduction

Ultraviolet (UV) radiation from the sun has adverse effects on human skin; it causes photoaging, sunburn and most seriously, skin cancer. The protective ozone layer has been subject to destruction and the areas where the ozone layer is thin have higher incidence of skin cancer.

Malignant melanoma is one of the most rapidly increasing types of cancer in Sweden and non-melanoma skin cancer is also increasing. UV-radiation from the sun is the most important etiological factor.

**Figure 1.**
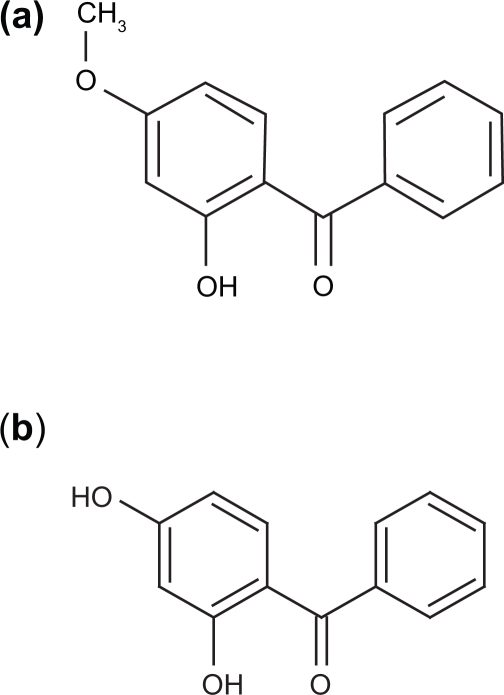
Chemical structure of BZ-3 (**a**) and DHB (**b**).

Sunscreens are widely used to protect us against harmful radiation. Benzophenone-3 (BZ-3) has been a commonly used filter in sunscreens for the last few decades; it has both UVA and UVB protecting properties. It is an organic chemical absorber with a molecule weight (MW) of 228.25 and CAS number 131-57-7. The structure is shown in Figure 1a (ChemIDplus). The ultimate sunscreen stays on the skin and its protective mechanism functions there. However, BZ-3 is relatively lipophilic (log P 3.64 ± 0.37) and several reports have shown that BZ-3 penetrates the skin and is excreted in urine (Hayden, Roberts et al. 1997; Gustavsson Gonzalez, Farbrot et al. 2002; Janjua, Mogensen et al. 2004; Sarveiya, Risk et al. 2004; Gonzalez, Farbrot et al. 2006). Urine is the major excretion pathway and 2,4-dihydroxybenzophenone (DHB), one of the major metabolites in rats (Okereke, Kadry et al. 1993). DHB has a MW of 214.22 and CAS number 131-56-6. The structure is shown in Figure 1b. BZ-3 is the most bioavailable compound of all UV-absorbing chemicals, (Nash, 2006) and is extensively conjugated in the human body.

We wanted to develop an assay to measure the amounts of BZ-3 and DHB in human urine. The method had to suit our needs. For example, it had to be possible to handle a large number of samples easily; hence it had to be rapid and simple but still sensitive, accurate and reproducible. Several methods using high performance liquid chromatography (HPLC) and BZ-3 have been described (Abdel-Nabi, Kadry et al. 1992; Jiang, Hayden et al. 1996; Vanquerp, Rodriguez et al. 1999; Chisvert, Pascual-Marti et al. 2001) and this work has been developed to some extent on the basis of previously described method (Abdel-Nabi, Kadry et al. 1992). However, there are few methods for extraction of BZ-3 and DHB in human urine after it has been metabolized by the human body (Sarveiya, Risk et al. 2004). Abdel-Nabi et al. used urine from rats (Abdel-Nabi, Kadry et al. 1992). Several other methods are designed for product evaluation and not for extraction from biological media. None of the methods fitted our needs completely and for that reason we developed this method. More than 1000 urine samples were collected and each was analyzed for conjugated/non-conjugated BZ-3 and conjugated/non-conjugated DHB. More than 4000 analyses were performed.

The method we developed was used to study the excretion pattern of BZ-3 and DHB in urine after repeated topical whole-body applications of a sunscreen containing BZ-3 to 25 human volunteers.

## Experimental

### Reagents

BZ-3 (2-hydroxy-4-methoxybenzophenone), DHB (2,4-dihydroxybenzophenone) and benzophenone (BZ), all purity 99%, (Aldrich Chem).

Methanol (HPLC-grade), acethonitrile (HPLC-grade) and trifluoroacetic acid (TFA) (Merck).

β-glucurunidase/arylsulfatase obtained from Helix pomata (Boehringer Mannheim).

### Chromatography

A gradient HPLC-system with a pump PU-1580 (Jasco), a 50 μl loop injector and a SPD-10A VP uv-vis detector (Schimadzu) were used. Chromatographic separation was achieved on a Genesis C18 ID (4.6 mm × 150 mm) column. A Genesis C18 (20 mm × 4.0 mm) was used as the precolumn.

The mobile phase was acethonitrile:dionized ultra filtered water (UF) (0.45 μm) 44:66 (v/v) with 1 mL TFA to 10000 mL mobile phase with gradient elution according to Table 1. UV absorption was done at 287 nm. The runtime was 31.5 min.

### Standard solutions

One stock solution containing 4.0 mmol L^−1^ BZ-3 and one stock solution containing 3.0 mmol L^−1^ DHB in 70:30 (v/v) methanol: UF water were used. Working standard solutions were prepared in methanol at five concentrations, 0.04 mmol L^−1^ BZ-3, 0.03 mmol L^−1^ DHB, 0.4 mmol L^−1^ BZ-3, 0.3 mmol L^−1^ DHB, 4 mmol L^−1^ BZ-3, 3 mmol L^−1^ DHB, 40 mmol L^−1^ BZ-3, 30 mmol L^−1^ DHB and 100 mmol L^−1^ BZ-3, 75 mmol L^−1^ DHB. All solutions were prepared in volumetric flasks, class A and kept refrigerated at temperature between 2 and 8 °C. Fresh standards were prepared every month.

### Sample pre-treatment

#### Pre-treatment before extracting conjugated samples

The urine samples were centrifuged at 2100 g for 10 minutes. 1 mL urine was used. 100 μL glucuronidase/arylsulphatase and 25 μL internal standard were added. The sample was incubated at 37 °C 16 hours. The sample was diluted with 2 mL phosphate buffer (50 mmol L^−1^, pH 6.5). Most samples were diluted 1:20 with 0.9% NaCl-solution.

#### Pre-treatment for non-conjugated samples

The urine samples were centrifuged at 2100 g for 10 minutes. 1 mL was used. 25 μL internal standard was added. The sample was diluted with 2 mL phosphate buffer.

### Solid-phase extraction

Extraction was performed with a vacuum manifold and solid-phase extraction (SPE) columns C8, 100 mg, 6 mL (Isolute Inc., purchased from Sorbent AB).

1. The column was activated with 2 mL methanol. The vacuum was turned off when the methanol reached the top of the sorbent, to prevent the column from drying. The procedure was repeated once.
2. The column was rinsed with 2 mL phosphate buffer. The vacuum was turned off when the buffer reached the top of the sorbent bed, to prevent column from drying. The procedure was repeated twice.
3. The sample was transferred to the column with a pasteur pipette and the sample was then drawn slowly through the column. The column was dried under full vacuum. The procedure was repeated once.
4. The column was rinsed twice with 1 mL phosphate buffer. After each rinse it was dried under full vacuum.
5. The sample was eluted four times with methanol: TFA 99:1 (v/v) 0,25 mL. After each eluation it was dried under full vacuum.
6. The samples were transferred to glass vials.

### Laboratory-made extraction controls

One healthy volunteer applied 2 mg cm^−2^ of a sunscreen containing 4% BZ-3, a total of 33 g of sunscreen containing 1.32 g of BZ-3. All urine was collected for 24 hours after the application. The urine was diluted with urine from four healthy volunteers into two different concentrations, 7.0 μmol L^−1^ and 45 μmol L^−1^ for BZ-3 and 0.86 μmol L^−1^ and 6.3 μmol L^−1^ for DHB.

### Internal standard

BZ was used as internal standard at the concentration 5 mmol L^−1^ (0.091 g). BZ was diluted in 100 mL methanol. Volumetric flasks, class A were used. The internal standard was prepared fresh every month and stored in a refrigerator at a temperature between 2 and 8 °C.

### Urine collection and urine samples

25 volunteers (16 women and 9 men; mean age 27 years, range 22–42) participated in the study. Height and weight were measured. Their body surface area (BSA) was calculated with the DuBois formula: BSA = 0.007184 × [height (cm)]0.725 × [weight (kg)]0.425). The sunscreen used was a commercially available sunscreen, SPF 14 containing 4% BZ-3. Before the first application of sunscreen each volunteer gave a urine sample to confirm that no BZ-3 was found in the urine prior to this investigation. Each volunteer received 2 mg cm^−2^ according to his or her BSA. The sunscreen was distributed in plastic containers, one for each application. The amount of sunscreen per application varied between the participants from 26 g to 47 g. The total amount of BZ-3 varied between 10.4 g to 18.8 g. BZ-3 was measured in the urine. One volunteer was excluded because the written instructions were not followed accurately.

The volunteers were instructed to apply the sunscreen evenly over the entire body, with the exception of the scalp and genital area, morning and night for 5 days, a total of 10 times. They were allowed one shower/day before the second application. During the five days the sunscreen was applied, all urine was collected, the volume measured and 10 mL from each sample saved and stored at −70 °C. Hence, each volunteer produced a different n umber of urine samples. They collected the urine for 5 days. After the last application they continued to collect urine for another 5 days, making a total of 10 days. The time of day, number and volume for each urine sample were recorded.

A total of 1234 urine samples were collected and analyzed.

## Results

### Minimum detectable limits

The minimum detectable limit was defined as three times the baseline noise level. The detection limits for BZ-3 and DHB were 0.01 μmol L^−1^ (0.1 ng per 0.05 mL sample) and 0.16 μmol L^−1^ (2 ng per 0.05 mL sample), respectively.

### Chromatography and selectivity

**Figure 2.**
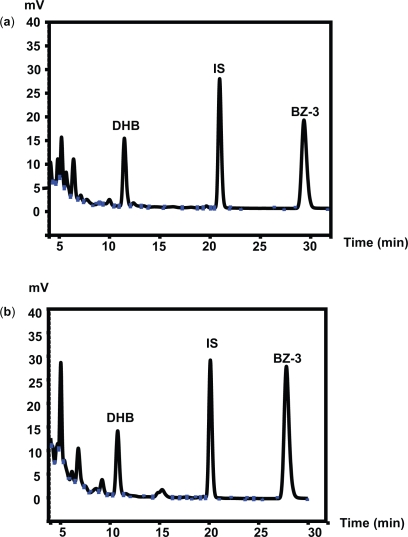
Chromatograms of conjugated BZ-3, conjugated DHB and IS. Chromatograms from subject 8 (**a**) and high concentration of laboratory-made extraction controls (**b**).

Figure 2 shows chromatograms of the HPLC-separation of BZ-3, DHB and internal standard.

It was more difficult to achieve separation for DHB since there was more interference in the beginning of the chromatogram.

### Calibration

Calibration was done against an external standard at five different concentrations 0.04, 0.4, 4, 40 and 100 μmol L^−1^ BZ-3 and 0.03, 0.3, 3, 30, 75 μmol L^−1^ DHB in a solution made of methanol and UF water.

A calibration curve was made for each sample series, which also consisted of extraction controls at low and high concentrations before each of the 9 urine samples. Each sample series consisted of 60 samples.

### Precision and linearity

Within-day precision showed relative standard deviations (RSD) 10% and 11% for the low concentrations of BZ-3 and DHB and 7.2% and 6.6% for the high concentration of BZ-3 and DHB, respectively. Between-days precision showed RSD 10% and 13% for the low concentrations of BZ-3 and DHB and 8.0% and 8.7% for the high concentration of BZ-3 and DHB respectively. There was no difference regarding precision between BZ-3 and DHB, except for the lowest concentration of DHB, which exhibited greater variation (13%) owing to interferences in the beginning of the chromatogram (Fig. 2).

BZ-3, DHB and internal standard showed excellent linearity with correlation coefficient (*r*^2^) > 0.99 for all concentrations.

### Excretion pattern

**Figure 3.**
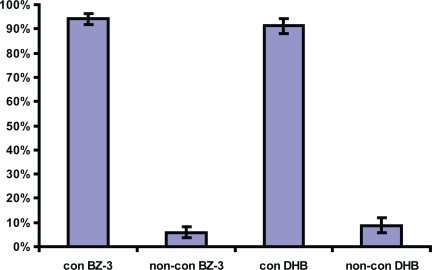
The relationships between conjugated/non-conjugated BZ-3 and conjugated/non-conjugated DHB. Both BZ-3 and DHB are extensively conjugated in the human body.

BZ-3 and DHB were extensively conjugated and only a small proportion was excreted in the non-conjugated form, mean values 5.9% and 8.8% respectively (Fig. 3).

**Figure 4.**
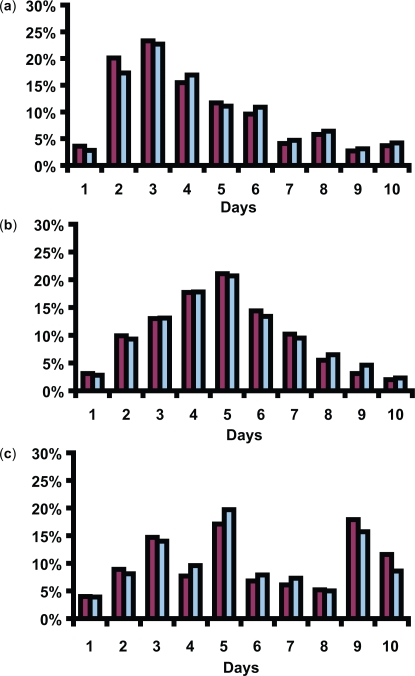
Excretion patterns of conjugated BZ-3 and conjugated DHB during the 10 days urine was collected. Regardless of pattern DHB showed a similar curve to BZ-3. Pattern 1 (9/24) shows rapid excretion on the first days and then evenly decreasing excretion (**a**). Pattern 2 (7/24) shows a slow and even increase followed by a decrease resembling a Gaussian curve (**b**). The third group did not fit into patterns 1 or 2 (8/24) (**c**).

The excretion pattern varied among the individuals. We discerned two patterns and three groups. Nine of the volunteers showed a pattern of rapid excretion of conjugated BZ-3 on the days when the sunscreen was being applied, followed by evenly decreasing excretion on the 5 consecutive days when the sunscreen was not being applied. This is exemplified in Figure 4a. Seven of the volunteers showed a slow and even increase of conjugated BZ-3 during the days the sunscreen was being applied and a slow and even decrease during the days the sunscreen was not applied, resembling a Gaussian curve. This is exemplified in Figure 4b.

Eight of the volunteers did not fit into any of the patterns above (Fig. 4c), forming a third group.

The excretion of conjugated DHB followed the excretion of conjugated BZ-3 regardless of pattern.

## Discussion

A total of 1234 samples were collected and each sample was analyzed for conjugated/non-conjugated BZ-3 and conjugated/non-conjugated DHB. More than 4000 samples were analyzed and we found this method very suitable for handling large numbers of samples.

Methanol/UF water was used instead of urine for the standard solutions because in high concentrations, the added BZ-3 was precipitated in the urine. For this reason we also included the laboratory-made extraction controls that where extracted with the SPE-columns in the same procedure as the other urine samples. They were also used to control the within-day and between-days variations, which were found to be sufficiently low for the method to be considered stable.

As internal standard BZ was used. It is a closely related structural analogue of BZ-3. The use of an internal standard provides a more correct quantitation.

The detection limits were at the same levels as in other methods, (Abdel-Nabi, Kadry et al. 1992; Sarveiya, Risk et al. 2004; Schakel, Kalsbeek et al. 2004) and they were sufficient to analyze the samples on day 10.

Previous studies in rats have shown that BZ-3 is extensively conjugated and excreted in the urine; although a small part is metabolized by cytochromes P-450 to DHB. DHB also undergoes extensive conjugation. A minor route of elimination of BZ-3 is faeces, which were not included in the present study. The metabolite 2,2′-dihydroxy-4-methoxybenzophenone (DHMB) was not investigated since it was detected only in trace amounts in the urine, but it was the major metabolite found in faeces (Okereke, Kadry et al. 1993).

2,3,4-trihydroxybenzophenone (THB) is also a metabolite of BZ-3. However, Sarvieya et al. have shown that THB is only found in trace amounts in human urine (Sarveiya, Risk et al. 2004). In our experimental set-up THB had unstable properties and it was therefore not analyzed.

Reverse-phase HPLC is a reliable method for detection and quantitation. Compared to other methods such as gas chromatography and mass spectronomy, reverse-phase HPLC is in general more available, most laboratories have access to this method.

Many endogenous substrates are also metabolized by conjugation, for example bilirubin and ethinylestradiol (Burchell, Brierley et al. 1995). In 1949 the grey baby syndrome was reported, caused by chloramphenicol toxicity in neonates due to their immature ability to conjugate (Sutherland, 1959). Strasbourg et al. have shown that for children between 13 and 24 months, the hepatic glucuronidation activity was lower for a number of drugs (Strassburg, Strassburg et al. 2002).

Pharmacogenetics is the study of how genes influence an individual’s response to drugs. There are several examples of when pharmacogenetics is of importance. In dermatology, hydroxychloroquine is used to treat severe cases of polymorphic light eruption and discoid lupus erythematosus. Inherited deficiency of glucose-6-phosphate dehydrogenase should be excluded before treatment with hydroxychloroquine is started.

There are also large inter-individual differences in the capacity to conjugate, for example 10% of the North American population is homozygous for the UGT1A1*28 allele. FDA has guidelines about the anti-cancer drug Irinotecan, stating that reduction in the starting dose should be considered for patients known to be homozygous for the UGT1A1*28 allele (FDA, 2005).

This could be one explanation why there are so pronounced differences in the excretion pattern between the subjects.

In the future the genotype might play a role in determining when to decide what sunscreen is best suited for an individual. If the person’s ability to conjugate is low, a sunscreen containing BZ-3 may not be the sunscreen of choice. There have been speculations about the estrogenic effects of some sunscreens, including BZ-3. It might not have any potent effect by itself but if a person is medicated with estrogens there might be a more potent effect from an endocrine active sunscreen in some individuals due to saturation of the conjugation enzymes. It seems that sunscreens containing BZ-3 are being phased out. For example, no sunscreens sold at the Swedish pharmacies contain BZ-3.

Future studies about genotype and sunscreens are needed to evaluate the importance of our findings.

## Figures and Tables

**Table 1.** Gradient program

	**Gradient program**				
**Time (min)**	0.1	16	18	30	31.5
**Flow (ml/min)**	1.0	1.0	1.9	1.9	1.0
